# Recent Advances and Current Challenges in Stereotactic Body Radiotherapy for Ultra-Central Lung Tumors

**DOI:** 10.3390/cancers16244135

**Published:** 2024-12-11

**Authors:** Viola Salvestrini, Andrea Lastrucci, Marco Banini, Mauro Loi, Maria Grazia Carnevale, Emanuela Olmetto, Pietro Garlatti, Gabriele Simontacchi, Giulio Francolini, Pierluigi Bonomo, Yannick Wandael, Isacco Desideri, Renzo Ricci, Daniele Giansanti, Vieri Scotti, Lorenzo Livi

**Affiliations:** 1 Radiation Oncology Unit, Oncology Department, Azienda Ospedaliero-Universitaria Careggi, 50134 Florence, Italy; viola.salvestrini@unifi.it (V.S.); andrea.lastrucci@unifi.it (A.L.); mauro.loi@unifi.it (M.L.); olmettoe@aou-careggi.toscana.it (E.O.); garlattip@aou-careggi.toscana.it (P.G.); gabriele.simontacchi@unifi.it (G.S.); francolinigiulio@gmail.com (G.F.); bonomop@aou-careggi.toscana.it (P.B.); vieri.scotti@unifi.it (V.S.); lorenzo.livi@unifi.it (L.L.); 2 Department of Allied Health Professions, Azienda Ospedaliero-Universitaria Careggi, 50134 Florence, Italy; wandaely@aou-careggi.toscana.it (Y.W.); riccire@aou-careggi.toscana.it (R.R.); 3 Department of Experimental and Clinical Biomedical Sciences “M Serio”, University of Florence, 50134 Florence, Italy; mariagcarne@gmail.com (M.G.C.); isacco.desideri@unifi.it (I.D.); 4 Centre TISP, Istituto Superiore di Sanità, 00161 Roma, Italy; daniele.giansanti@iss.it

**Keywords:** SBRT, ultra-central, NSCLC, oligometastasis, oligoprogression

## Abstract

Stereotactic body radiotherapy has been established as a viable treatment option for inoperable early-stage non-small cell lung cancer or secondary lesions mainly in oligoprogressive/oligometastatic scenarios. Treating lesions in the so-called “no flight zone” has always been challenging and conflicting data never cleared how to safely treat these lesions. This is truer considering ultra-central lesions, i.e., directly abutting or whose PTV is overlapping critical mediastinal organs. While historical retrospective data are abundant but mostly heterogenous in terms of the definition of ultra-central lesions, dosing regimens and outcomes, prospective data remain scarce, even though recently published studies have given new encouraging results for such delicate treatment scenarios. For this reason, we aimed to review and summarize current knowledge on stereotactic radiation treatment for ultra-central thoracic lesions, highlighting the most recent advances and the messages that can be taken from them. Lastly, we propose a workflow of the necessary steps to identify and treat such patients, therefore helping in elucidating the advantages and caveats of such treatment options.

## Simple Summary

Stereotactic radiotherapy for ultra-centrally located primary or secondary thoracic lesions has always been challenging if not thought to be too harmful for patients when applied with ablating purposes. Recent prospective evidence has reopened this possibility though some warning alerts have come out from other experiences. The SUNSET trial showed that 60 Gy/8 fractions radiotherapy is feasible with an acceptable toxicity rate, but other studies such as LUNGART and HILUS trial suggest that appropriate patient selection and follow-up is of utmost importance. The present critical review aims to summarize the current state of the art in stereotactic radiotherapy for ultra-centrally located lesions and propose a practical workflow that may aid the radiotherapy community in a critical discussion and offer of such treatment with their patients.

## 1. Introduction

For patients with early-stage NSCLC who are deemed inoperable, stereotactic body radiotherapy (SBRT) has been established as a viable treatment option [1,2,3,4,5,6], yielding comparable survival rates and lower risk of treatment-related mortality than surgery and better OS than conventional radiotherapy (RT) [7]. Furthermore, SBRT is currently one of the most widely adopted options for metastasis-directed therapy (MDT) in the setting of oligometastatic/oligoprogressive disease [8,9]. 

Indeed, SBRT allows the delivery of high doses of radiation per fraction, with steep dose gradients, by multiple co-planar and non-coplanar beams, and guided by a set of coordinates. To achieve an ablative effect, dose schedules must be equivalent to a biologically effective dose (BED) of at least 100 Gy: accumulating evidence shows that infield control exceeds 85% and grade 3 toxicities are rare (<5%) [2,10]. However, most of the available literature on SBRT outcomes in lung lesions comes from the treatment of peripherally located tumors, whereas for centrally located tumors, the use of SBRT is still debated [11,12,13,14,15,16,17]. In this scenario, Timmerman et al. showed that hilar and perihilar tumors treated with SBRT to 60–66 Gy in three fractions had an 11-fold increased risk of severe toxicity and 2-year freedom from severe events of 54% compared with 83% of peripheral tumors, thus defining a new category of “central” lesions, i.e., located within 2 cm of the proximal bronchial tree (PBT), in the so-called “no-fly zone”, where moderate hypofractionation is recommended [11]. Yet, in the pivotal RTOG 0813 dose-escalation study, the five-fraction regimen with a maximum of 12 Gy/fraction proved to be safe, with a grade 3–4 toxicity of 7% and a 2-year local control (LC) rate of 88% in centrally located NSCLC stage I patients [18]. However, selecting risk-adapted schedules of SBRT (RT), some experiences have demonstrated that RT can be safely administered also to a subset of central tumors that abut the central airway, esophagus, or other mediastinal structures, “ultra-central” (UC) tumors [10,11,12]. A recent systematic review and meta-analysis of this population confirmed a pooled incidence of grade 3–4 toxicity events of 6%, most commonly pneumonitis, after SBRT [19]. Although no increased toxicity was reported in the 50 Gy in the 4–5 fraction retrospective study, other studies investigating UC lung tumors have reported grades 3–5 in 30% or more, raising concerns about the potential risk of fatal complications [20,21,22]. While a dose of PBT did not increase toxicity rates, esophageal injury, such as esophageal fistula, and radiation pneumonitis were related to maxi parameters. Specifically, higher AE rates and mum esophageal dose and mean lung dose, respectively [23]. To this day, the literature is still contradictory and mostly based on retrospective data. Hence, the definition of UC tumors and the role of SBRT in this setting is still an unanswered question and often debated. Therefore, we performed an overview of the recent advances and current challenges in SBRT for UC lung tumors. We speculate that analyzing the general and technical requirements for prescription and dosimetric constraints could contribute to SBRT being administered safely and effectively for UC lung tumors.

## 2. Definition of Ultra-Central Tumors

The ultra-central definition was first introduced by Chaudhuri et al. in 2015 to indicate a subset of central tumors directly abutting the central airway (i.e., trachea and PBT) [24].

Other authors have further broadened its meaning, including in the classification of UC also those lesions with a PTV overlapping with other hilar structures (i.e., esophagus or pulmonary vessels.) [20,25,26]. However, different definitions (Table 1) have been introduced by the retrospective cohorts of UC tumors, pointing out the lack of consensus among the experts. The HILUS phase II trial defined UC tumors as lesions located 1 cm zone around the carina, main bronchi, intermedius bronchus, and lobar bronchi [27]. Some authors considered planning tumor volume (PTV) and others the gross tumor volume (GTV) approaching the organ at risk (OAR). The most commonly recognized UC regions of interest are PBT, trachea and esophagus but also heart, pulmonary vein and artery are cautionally included in some definitions. The demanding dosimetric constraints recommended for the central airways and esophagus might justify this selection and the correlated risk of severe complications after SBRT of UC lung tumors. According to the meta-analysis by Yan and colleagues [19], all studies included the overlap of PTV with the PBT in their definitions, while only 59% considered GTV. The PTV overlap with other mediastinal structures, including the great vessels and esophagus, was reported in 52% of articles, whereas only four studies permitted direct contact of the GTV with these OARs [19].

**Table 1:** Summary of main findings from studies on treatment of ultra-central lung tumors.

First Author and Year [Reference]	Sample Size (Treatment Years)	Type	Definition of Ultra-Central	Primary Lung Cancer pts	Met pts	Fractions × Dose per Fraction	Median Tumor Max D or GTV Volume	Median FuP (mo)	SBRT Technique	Main Results and Comments
Swaminath, 2024 [28]	23 pts (2014–2020)	Phase III (RCT conventional RT vs. SBRT, not stratified for UC tumors)	Tumors abutting PBT or mediastinal organs	23 (100%)	0	8 × 7.5 Gy	25 mm (general population)	36.1	3DCRT, VMAT, IMRT, CK allowed	3-ys LC 87.6%, EFS 49.1%, OS 63.5% (overall SBRT population). 1 (4.3%) late (12 months) G5 hemoptysis in a tumor abutting proximal bronchus. 4 (17.4%) G ≥ 3 TRAEs. No dosimetry issues according to protocol were found in G5 event.
Giuliani, 2024 [29]	30 pts (2018–2021)	Phase I	PTV touches or overlaps the central bronchial tree, esophagus, pulmonary vein, or pulmonary artery	30 (100%)	0	8 × 7.5	26 mm	36	3DCRT, VMAT, CK allowed	3-ys OS 72.5%, PFS 66.1%, LC 89.6%, RC 96.4%, and DC 85.9%. 6.7% G3–5 TRAEs: 1 G3 dyspnea and 1 G5 pneumonia. PTV Dmax limited to 120%; tumors with endobronchial invasion were excluded.
Levy, 2024 [30]	6/31 pts UC (2015–2017)	Phase II	GTV ≤ 1 cm from trachea or mainstem bronchi; central: ≤2 cm from PTB or immediately adjacent to pericardial or mediastinal pleura	6 (100%)	0	8 × 7.5	26 mm	43	IMRT, VMAT, Tomotherapy allowed	3-ys cumulative rate of LP 6.7%. 3-ys PFS and OS 81.5% and 61.1%. 16.1% G ≥ 3 and 3.2% G5 (pneumonitis) early AEs. 58.1% G ≥ 3 and 3.2% G5 (hemoptysis after bronchoscopy) late AEs.
Rim, 2024 [31]	20 (2017–2021)	Retrospective	Tumor abutting or invading PTB.	20 (100%); 2 recurrents, 1 SCLC.	0	10 × 4.5 (5%) 10 × 5–6 (95%)	35 mm	15.8	IMRT, VMAT	1-y and 2-ys OS rates were 79.4% and 62.4%, 1-y and 2-ys LC rates were 87.1% and 76.2%. 1 (5%) G ≥ 3 AE = G5 hemoptisis (patient with endobronchial involvement) = 5%. Dmax < 110%
Bryant, 2024 [32]	14 (2019–2021)	Retrospective	GTV ≤ 1 cm from trachea or mainstem bronchi	9 (64.3%)	5 (35.7%)	8 × 7.5 Gy	17.8 cc	17.2	IMRT MRI-guided	2-ys LC, LFFS, OS, and PFS rates were 92.9%, 85.7%, 92.9%, and 64.3% No acute or late G ≥ 3 AEs. Adaptive plan permitted PBT Dmax of 5.7 Gy and GTV D95% at 99.8%. Hotspots ≤ 120%.
Li, 2024 [33]	154 (2009–2019)	Retrospective	PTV abutting or overlapping central bronchial tree or esophagus	32 (20%) treated in curative setting	122 (80%)	5 × 10 most common (42%) 5 × 6–11 (median 9)	27 mm	21.5	IMRT, VMAT	mOS 44 months, mPFS 8.8 months. 3-ys LC 86%. G3 acute AEs = 3%, 2 esophagitis, 1 atrial fibrillation, 1 pericarditis, 1 pleural effusion. G ≥ 3 late AEs = 4.9%, 3 G3 pneumonitis, 1 G3 chest wall pain, 1 G3 bronchopleural fistula; 1 G4 esophagitis, 1 G4 bronchial obstruction; 1 G5 pneumonitis. Tumor volume overlapping with esophagus correlated with worse LC. Predictors of severe toxicity = PTV size, decreased PTV V95%, lung V5 Gy, and lung V20 Gy.
Lee, 2024 [34]	19 (2019–2022)	Retrospective	GTV abutting PTB, esophagus or great vessels. GTV ≤ 2 cm from PTB and mediastinum considered central.	0	19 (100%)	5 × 7–12 (median 10)	NR	19	IMRT MRI-guided adaptive RT	1-y and 2-ys LC was 94% and 86%. Median time to distant recurrence 6.6 months. 32% G2 acute toxicities, no other AEs. Plan adapted with isotoxic approach. Re-optimization showed better PTV coverage than original plan. 85% patients had immune and TKI therapy < 1 months before SBRT. VEGFRi held >4 weeks before.
Ahmadsei, 2023 [35]	60 (2014–2021)	Retrospective	PTV overlapping or abutting the PBT, trachea or esophagus	27 (45%)	33 (55%)	8 × 5–6 Gy 10 × 4.5–5 Gy	30–70 mm for 66.7% patients.	26.4	VMAT	2-ys OS 65.9% 1-y and 2-ys LC 84.4% and 76.8% 2-ys DC 45% 3% G ≥ 3 Aes: 1 G3 and G4 pneumonitis. 20% cardiovascular events at 2 years: 10% valvopathy, 8.3% atrial fibrillation. Hypothetic association between dose to pulmonary artery and superior cava vein and non-cancer-related deaths. No other cardiac substructures dosimetry concerns.
Iovoli, 2023 [36]	49/93 UC pts (2007–2021)	Retrospective	Directly abutting any of proximal airway, mediastinum, great vessels, spinal cord. ≤2 cm categorized as central	93 (100%)	0	5 × 10–12 Gy	NR	32.4	3DCRT, VMAT	SAN Dmax and Dmean significantly associated with worse OS (*p* = 0.026 and *p* = 0.011, respectively), with cut-off values of 1309 cGy and 836 cGy.
Lindberg, 2023 [37]	230 pts/238 lesions (2010–2018)	Phase II (65 pts) + retrospective studies (165 pts)	UC (groups A,B,D) 1 cm zone around the carina, main bronchi, intermedius bronchus, and lobar bronchi (i.e., the PBT) C (group C): 1–2 cm around the PBT	196 (77%)	54 (23%)	8 × 7 Gy	35 mm	24 (phase II studies, nr for overall cohort)	VMAT	1-y, 3-ys and 5-ys LC rates at were 92%, 84% and 78%. 1-y, 3-ys and 5-ys OS rates were 78%, 40% and 27%. G 3–4 toxicity in 15% pts, and 13% (30 pts) had G5 tox (20 hemoptysis, 7 pneumonia, 2 cardiac failures, 1 COPD). Tumor compression of PBT and high maximum dose to the mainstem or intermediate bronchus increased the risk of fatal toxicity.
Song, 2023 [38]	27 pts (2013–2018)	Retrospective	PTV touching or overlapping the central bronchial tree, esophagus, or pulmonary artery	27 (100%); 4 recurrent	0	10 × 6 Gy 7 × 8 Gy	37 mm	41	IMRT	mOS and mPFS 48 months and 36 months. G ≥ 3 AEs in 5 pts (18.5%): 1 G3 pneumonitis, 2 G3 bronchial obstructions, 1 G5 bronchial obstruction, 1 G5 esophageal perforation. No difference in outcomes, but higher toxicity when compared to analogous central tumors studies (G3 = 0), with higher Dmax to lungs, bronchus, esophagus and heart.
Tonneau, 2023 [39]	65 lesions (2009–2019)	Retrospective	PTV touching or overlapping the central bronchial tree, esophagus, pulmonary vein, or pulmonary artery.	65 (100%)	0	Mostly 5 × 10 Gy	NR	37.6	VMAT, Cyberkinfe, Tomotherapy	After 37.6 months median follow-up: 10% LR, mOS 37.3 months and mDFS 36.6 months. 2 G5 TRAEs: pneumonitis. Comparison with central and peripheral pts from same center: higher RR e DR with UC lesions (CHR 2.44, 2.15); shorter OS and PFS versus central and peripheral lesions. BED10 < 120 correlated with higher LR, RR e DR risks.
Tekatli, 2023 [40]	94 pts (2008–2015)	Retrospective	GTV ≤ 1 cm from PBT	94 (100%)	0	8 × 7.5 Gy 12 × 5 Gy	44 mm	40.5	VMAT	Considering additional 33 C lesions: mOS 25 months; 3-ys and 5-ys LC 78% and 69%; 3-ys and 5-ys RC 81% and 72%. G ≥ 3 AEs = 20% of which 21% pulmonary, 1% bone fracture. G5 = 12%, all pulmonary, mostly >12 months. Location ≤ 1 cm from trachea or bronchus and PS 2–3 correlated with pulmonary toxicity.
Regnery, 2023 [41]	16 patients/16 lesions 2020–2021	Prospective database	PTV overlapping with the PBT or esophagus	4 (25%)	12 (75%)	12 × 5 Gy 10 × 5.0–6 Gy 8 × 7.5 Gy 8 × 5 Gy 6 × 5 Gy 5 × 6 Gy	NR	24	IMRT MRI guided adaptive RT	2-ys OS 67%, 2-ys PFS 37%, 2-ys LC 93%. AEs G ≥ 2 = 56%, 1 G3 bronchial bleeding, 1 G4 bronchial bleeding (further treated with VEGFRi), 1 G3 esophagitis. Lowest BED fractionations used for tumors abutting esophagus. Comparison with C tumors treated with MRI-IMRT: Higher AE rates but no difference in outcomes.
Sandoval, 2023 [42]	38/47 ultra-central patients (2019–2021)	Retrospective	GTV ≤ 1 cm from trachea, mainstem bronchi or PBT. C lesions defined as ≤2 cm from PBT, mediastinum or pericardium	22 (46.8%)	25 (53.2%)	3 × 18 (3.5%) 5 × 10–12 (25.6%) 8 × 7.5 (47%) 10 × 5 (6%) 15 × 4 (17.9%)	NR	22.9	IMRT-MRI guided adaptive RT	1-y LC 87% (median NR), 1-y OS was 82% (median NR), 1-y PFS was 54%. No acute G ≥ 3 toxicity, 5% late G3 toxicities: esophagitis and pneumonitis. G2 toxicity associated with GTV volume. No statistical outcome differences between UC vs. non-UC lesions.
Rock 2023 [43]	50 patients (2009–2020)	Retrospective	PTV overlap or direct tumor abutment with the major vessels, esophagus, or central airway	34 (68%)	16 (32%)	10 × 4–7 Gy (median 6.5)	NR	13 (range 0.3–102)	3DCRT; IMRT; VMAT	Primary NSCLC: 1-y LC = 83.8%, 3-ys LC = 65.4%; 1-y PFS = 50.1%, 3-ys PFS = 26.8%; 1-y OS = 93.7%, 3-ys OS = 70.5%. Oligometastatic: 1-y LC = 85.2%; 1-y PFS = 12.5%, 1-y OS = 88.9%, 3-ys OS = 44.4%. G ≥ 2 AEs = 22%: 12% G2 pneumonitis, 2% G3 pneumonitis, 2% G2 airway obstruction, 4% G3 obstruction, 2% G5 hemoptysis.
Hiroshima, 2022 [44]	16 patients (2017–2020)	Retrospective	Within 2 cm within the PBT	16 (100%)	0	10 × 6 Gy 4 × 13.75 Gy	NR	14.4	IMRT or VMAT (1–4 fiducials; 4D CT scan)	No LR. OS, cancer-specific survival and PFS at 2 ys: 54.6%, 85.1%, and 33.7% 1 G3 radiation pneumonitis (no other G ≥ 3 Aes).
Ligtenberg, 2022 [45]	12 patients, (2017–2019)	Retrospective	Proximity to the mediastinum	12 (100%)	0	8 × 7.5 Gy	NR	NR	IMRT or VMAT	MidP-based treatment yield lower OAR doses compared to ITV-based treatment plans on the MR-linac (Mean lung dose significantly lower, difference: −0.3 Gy; *p* < 0.042).
Farrugia, 2022 [46]	83 patients, (2010–2019)	Retrospective	C: <2 cm within the proximal airway, mediastinum, great vessels, or spinal cord; UC: directly abutting any of the above structures	83 (100%)	0	5 × 10 Gy; 5 × 11 Gy.	<20 mm 68.7%	33.4	3DCRT/ VMAT	At log rank test and MVA, D45% right atria constraint (candidate cutoff values of 890 cGy) was significantly associated with non-cancer associated survival and overall survival (*p* = 0.0019 and 0.0044, respectively).
Salvestrini, 2022 [47]	122 pts/126 lesions (2006/2020)	Retrospective	PTV touches or overlaps the trachea, mainstem-, intermediate-, upper-, middle- or lower- lobe bronchus or the esophagus	68 (54%)	58 (46%)	7 × 7–8 Gy 6 × 8 Gy 5 × 9–12 Gy	37.5 mm	23	Cyberknife	1-, 2-, and 5- ys OS rates were 75%, 58%, and 23% 1-, 2- and 5-ys PFS rates at were 63%, 41%, and 15% 1-, 2-, and 5-ys LC rates were 86%, 78%, and 61%. Acute G2 dysphagia, cough, and dyspnea were 11%, 5%, 3%. Acute G3 dyspnea was 0.8%. Late G3 AEs rate = 4%. Tumor size and location close to the trachea rather than PBT correlated with better OS.
Wang, 2022 [48]	58 pts (2010–2018)	Retrospective	PTV touching or overlapping the PBT, trachea, esophagus, heart, pulmonary vein, or pulmonary artery within 2 cm around the bronchial tree in all directions	58 (100%)	0	7 × 8 Gy, 8 × 7 Gy, 6 × 9.3 Gy	NR	57	Cyberknife	1-, 2- and 5-ys OS rates were 94.7%, 75.0%, and 45.0%. 1-, 2- and 5-ys LC rates were 91.5%, 78.0%, and 58.6%. G ≥ 3 Aes = 3.5%. Pts with PTV < 53.0 cc = better OS.
Guillaume, 2021 [49]	74 pts/74 lesions (2012–2018)	Retrospective	PTV overlapped one of the following OARs: the trachea, right and left main bronchi, intermediate bronchus, lobe bronchi, oesophagus, heart.	37 (50%)	37 (50%)	5–10 × (4.5–10 Gy)	18.3 cc	25	CyberKnife, VMAT	1-y LC rate 96.7%, 2-ys LC rate 87.6% mPFS 12 months. mOS 31 months. G3 AEs = 2.7%. No G4–5 AEs. The type of OAR overlapping with PTV did not relate to AE risk. LR more common with GTV receiving Dmin BED10 ≤ 50 Gy (*p* = 0.002).
Farrugia, 2021 [50]	43 pts (2010–2019)	Retrospective	GTV abutting the proximal bronchial tree, trachea, mediastinum, aorta, or spinal cord.	43 (100%)	0	5 × (10–11 Gy)	12.4 cc	29	3DCRT/ VMAT	UC location was associated with worse non-cancer associated survival and OS, supposedly due to excessive D4 cc (of 18 Gy) dose to the proximal airways.
Breen, 2021 [51]	110 pts (2008–2019)	Retrospective	GTV directly touching the PBT or trachea. (2) PTV overlapping the trachea or mainstem bronchi.GTV within 1 cm of the PBT.	110 (100%)	0	4–8 × (7.5–12 Gy) (no 7 fractions)	17.7cc	30	3DCRT, VMAT	OS at 1, 2, and 5 ys was 78%, 57%, and 32% Local progression at 1, 2, and 5 ys was 4%, 16%, and 21%. Acute and late grade 2 + toxicity was seen in 18% and 27%. Four patients (4%) had fatal toxicity.
Lodeweges, 2021 [52]	72 pts (2012–2020)	Retrospective	PTV abutting or overlapping the main bronchi, trachea and/or esophagus	72 (100%)	0	12 × 5 Gy	NR	19	VMAT	3-ys and 2-ys LC rates were 98% and 85%. OS rates at 1- and 2-ys were 77% and 52%. G ≥ 3 was observed in 21%, of which 10 patients (14%) with G ≥ 5 bronchopulmonary hemorrhage. grade > 3 toxicity found correlated with Dmean to the main bronchus (*p* = 0.003), with cutoff value of BED3 = 91 Gy.
Mihai, 2021 [53]	57 pts (2008–2016)	Retrospective	(GTV) abutting or involving trachea, main or lobar bronchi.	37 (65%)	20 (35%)	4–10 × (5–12 Gy) (no 7 fractions)	NR	26.5	IMRT	mOS was 34.3. Freedom from local progression at 2 and 4 years was 92 and 79.8%. Fatal hemoptysis 8.7%.
Regnery, 2021 [54]	51 pts (2012–2019)	Retrospective	Overlap of the PTV with the PBT	37 (72.5%)	14 (27.5%)	10 × 5 Gy	NR	NR	3D, helical Tomotherapy, or VMAT	2-ys local failure rate UC = 26.9%; C = 14.6%. 2-ys OS C = 55.4%; UC = 54.9%. 2-ys AE G ≥ 3 15.3% for UC and 7.3% for C lesions. No grade 4 toxicity and only 1 potential grade 5 tox in UC cohort.
Cooke, 2020 [55]	27 pts	Retrospective	NR	0	22 (81%)	6 × 10 Gy (no 7 fractions)	6.6 cc	11.6	IMRT, VMAT	1-year OS 82.7 2-year OS 69.5 1-year IFC 95.2% 2-year IFC 85.7% No AEs G > 3
Loi, 2020 [56]	109 pts (NR)	Retrospective	PTV overlapping with central bronchial tree, esophagus, pulmonary vein, or pulmonary artery	0	109 (100%)	5–10 × (6–10) Gy	60 cc	17	VMAT	2-ys LC 87%. Improved LC was correlated to PTV V95% > 85% and to GTV < 90 cc. Overall and G ≥ 3 toxicity incidence was 20% and 5%, respectively.
Shahi, 2020 [57]	52 pts (84 mets) (2014–2019)	Retrospective	NR	0	52 (100%)	5 × (6–10) Gy	20 mm	20	VMAT	2-ys Local failure was 9.0%. Median PFS was 4.0 months, and median OS was 31.7 months. AEs G ≥ 3 in 6 (11.5%) pts, 71% transient. There was a single (1.9%) G 5 toxicity (radiation pneumonitis).
Wang, 2020 [23]	88 pts (2008–2017)	Retrospective	GTV abutting the proximal bronchial tree or PTV overlapping esophagus	53 (60%)	35 (40%)	5 × 9–10 Gy 8 × 7.5 Gy 15 × 4 Gy	NR	19.5	IMRT, VMAT	1 and 2-ys rates of local failure were 12.2% and 19.0%. 1, 2 and 3-ys OS rates for pts with primary NSCLC were 78.6%, 64.5% and 53.1%. AEs G ≥ 3 22%, including 6 (7%) G ≥ 3 radiation pneumonitis and 4 (4%) G ≥ 3 esophageal injury. TRAEs G5 in ten pts (11.4%) = hemoptysis, radiation pneumonitis, respiratory failure. BED10 ≥ 100 did not correlate with local control (UVA); lung V20 correlated with G ≥ 2 pneumonitis, not dose to PBT; Dmax, D2.5cc, D5cc to esophagus correlated with G ≥ 3 esophageal AE.
Zhao, 2020 [58]	98 (2013–2017)	Retrospective	PTV overlapping with PBT, esophagus, pulmonary vein or pulmonary artery	76 (77.6%)	22 (22.4%)	8 × 7.5 Gy	NR	22.9	3DCRT, IMRT or VMAT	2-ys and 3-ys LC, 97.8 and 84.5%. AEs G3 = 3 in the C group (2 dyspnea, 1 pneumonitis) and 2 in the UC group (1 dyspnea, 1 hemoptysis). No G > 3 toxicities. ITV predictor for LC (*p* = 0.001).
Yang, 2020 [59]	21 patients, 2012–2018	Retrospective	UC: PTV abutting or overlapping central structures (including PBT, heart, and great vessels but not the esophagus)	21 (100%)	0	8 × 7.5 Gy	NR	15	VMAT	The 1- and 2-ys OS rates were 87.5% and 76.6%. The 1- and 2-ys PFS rates were 71.1% and 64.0%. The 1- and 2-ys LC rates were 92.9% and 92.9%. AEs G2 19.1%. No G ≥ 3.
Duijm, 2019 [60]	188 patients, 2012–2016	Retrospective	UC: GTV ≤ 2 cm of the esophagus, trachea, mainstem, intermediate, upper, middle or lower lobe bronchus	154 (82%)	34 (18%)	8 × 7.5 Gy; 12 × 5 Gy	36 mm	NR	VMAT	Acute AEs: G1 (*n* = 19) and G2 (*n* = 10) only. Late AEs: 2 possible treatment-related deaths and 2 G3. DVH significantly correlated to acute and late AEs (*p* < 0.001).
Meng, 2019 [61]	80 patients, 2006–2015	Retrospective	C: GTV < 2 cm of, but not abutting, the PBT UC: GTV abutting the PBT	80 patients (100%)	0	10 × 6 Gy (C); 7 × 8 Gy (UC)	NR	44.5	CK	UC tumors showed worse OS, PFS, and LC compared to C lesions. On MVA, UC and PTV were poor prognostic factors. Toxicity profile similar in the two groups (UC vs. C).
Cong, 2019 [62]	51 patients, 2014–2017	Retrospective	UC: GTV abutting or over-lapping the trachea or PBT	51 patients (100%)	0	5 × 7 Gy	68 mm	17	CK	Median LC was 17 months for stage III pts and 11 months for stage IV or recurrent pts. G3 radiation pneumonitis was recorded in 3 pts (5.9%) and possible treatment-related death in 2 pts (3.9%).
Bezjak, 2019 [18]	120 patients (100 pts PP analysis, 17 UC), (2009–2013)	Prospective, phase I/II study	C: GTV < 2 cm around the PBT or immediately adjacent to the mediastinal or pericardial pleura	120 (100%) (100 pts PP analysis)	0	q 2 day fractionation × 5 fractions over 1.5–2 weeks: Dose Level 1: 5 × 8 Gy 2: 5 × 8.5 Gy 3: 5 × 9 Gy 4: 5 × 9.5 Gy 5: 5 × 10 Gy 6: 5 × 10.5 Gy 7: 5 × 11 Gy 8: 5 × 11.5 Gy 9: 5 × 12 Gy Protocol treatment begins at Level 5. Levels 1–4 employed if DLT is seen with the Level 5	11.2 cc	37.2	3DCRT; VMAT; IMRT	MTD was 12.0 Gy/fx, with a probability of a DLT of 7.2%. 2-year LC rate in this cohort was 87.9%. 2-year PFS in this arm was 54.5%. 2-year OS was 72.7%. Four pts (12.1%) experienced G3 AE during the first year; 1 pts (3%) reported G5 toxicity > 1 year.
Nguyen, 2019 [63]	68 patients, (2009–2017)	Retrospective	C: PTV < 2 cm of the PBT UC: PTV overlapped the PBT or esophagus	53 (78%)	15 (22%)	8 × 5 Gy 5 × 8 Gy; 5 × 10 Gy; 4 × 12.5 Gy; 5 × 11 Gy; 8 × 7 Gy; 8 × 7.5 Gy; 5 × 12 Gy.	NR	19.7	IMRT; VMAT	The 2-year estimates of LC (89% and 85%; *p* = 0.72) and OS (76% and 73%; *p* = 0.75) for UC and C tumors were similar. UC tumors increased risk of G2 tox (57.6% vs. 14.2%; *p* = 0.007) at 2 years. One patient with an UC tumor developed G5 respiratory failure.

Abbreviation: UC, ultra-central; C, central; LC, local control; OS, overall survival; G, grade; pts, patients; EFS, event-free survival; PFS, progression-free survival; NR, not reported; AE, adverse events; TRAE, treatment-related adverse events; NSCLC, non-small-cell lung cancer; DVH, dose-volume histogram; MTD, maximum tolerated dose; DLT, dose limiting toxicities; MVA, multivariate analysis; PTV, planning target volume; ITV, internal target volume; OAR, organ at risk; tox, toxicity; IMRT, intensity modulated radiation therapy; VMAT, volumetric modulated arc therapy; CK, Cyberknife; 3DCRT, three-dimensional conformal Radiation therapy; SAN sinoatrial node; LR, local recurrence; RR, regional recurrence; DR, distant recurrence; MRI, magnetic resonance imaging; MidP, mid position.

## 3. State of the Art for SBRT for Ultra-Central Tumors

To better describe the actual state of the art for SBRT in UC thoracic lesions, we conducted a critical review of the available data, searching through Medline, EMBASE and Google Scholar for published articles reporting a specific research string (hypofractionated[tw] OR stereotactic[tw] OR SABR[tw] OR SBRT[tw] OR radiosurgery[mh] AND (ultracentral*[tw] OR ultracentral*[tw] OR ultra-central*[tw] OR central*[tw]) AND (lung[tw] OR thorac*[tw] OR pulmonary[tw] OR lung neoplasms[mh]) and a time span between 1 January 2019 to 9 February 2022. Among the 301 results, 105 articles were selected by titles. We excluded (1) retrospective case studies of fewer than 10 patients, (2) studies of central lesions only or mixed for which no UC data were retrievable, (3) papers regarding only quality assurance or dosimetry calculations and (4) articles regarding SBRT to other disease sites. Hence, we included 38 articles whose authors, year of publication, definition of UC applied, design, dose fractionation and main findings are summarized in Table 1. We adopted 1 January 2019 as a starting point for our research due to the systematic review published by Chen H and colleagues [64]. Since 2019, only 6 prospective studies including UC lesions treated with SBRT results were published, while all the rest of the data are provided by retrospective studies.

### 3.1. Survival Outcomes and Toxicity with SBRT for Ultra-Central Tumors: Prospective Data

The prospective studies published so far enrolled a maximum of roughly 30 patients with UC thoracic lesions in dedicated or mixed cohorts. In the first historical phase I/II study RTOG 0813 trial, 2-year LC, PFS and OS rates of 87.9%, 54.5% and 72.7%, respectively, were reported in patients treated with 60 Gy/5 fx dose level (deemed to be the maximum tolerated dose within the study), even though most UC patients were treated within the 57.5 Gy/5 fx level. Treatments were delivered with IMRT, VMAT or 3DCRT. Notably, within the highest dose level (60 Gy/5 fx), only one patient reported a late G5 event (3%) [18].

In 2023, an expanded group analysis of the HILUS trial was published, confirming the warning points previously highlighted from the original prospective phase II study, which enrolled a total of 65 patients. In order to report outcomes coming from a larger population, the authors added a retrospective study of 165 UC patients (defined as per HILUS protocol within groups A, B, D) that were all treated with 56 Gy/8 fx schema. Control and OS rates were aligned with an RTOG study, with 1-year, 3-year and 5-year LC and OS rates at 92%, 84%, 78% and 78%, 40%, 27% (to be noted that patients with unresectable early stage or metastatic NSCLC were included), respectively. The warning sign coming from this study when treating UC tumors with SBRT is the treatment-related toxicity rates reported with 30 (13%) fatal toxicities: 20 hemoptysis, 7 pneumonitis, 2 cardiac failures and 1 COPD. Authors also found out that treating a tumor compressing the PBT and the Dmax to mainstem or intermediate bronchi was correlated with a higher risk of fatal toxicity and for this reason they eventually suggested that dose constraints used for main bronchi should be applied to intermediate bronchi as well when treating such high-risk lesions [37].

More prospective data raising concerns on the treatment of UC lesions come from the recently published Lungtech trial. The trial was closed early due to poor recruitment; enrollment was slowed due to suspensions as a result of fatal AEs. Thirty-one patients with centrally located tumors including six patients with UC primary NSCLC were treated per protocol with IMRT, VMAT or Tomotherapy at 60 Gy/8 fx dose. Survival outcomes were favorable for tumor control and overall survival (OS), with 3-year cumulative local recurrence at 6.7%, 3-year OS and PFS at 61.1% and 81.5%. Toxicity rates were once again concerning, with early G ≥ 3 AEs at 16.1% and one fatal pneumonitis and late G ≥ 3 AEs rate at 58.1% (mainly pulmonary): one fatal hemoptysis occurred after a procedural bronchoscopy. Even though not strictly treatment-correlated, other five late fatal AEs were also reported (for a total of 19.4% reported G5 AEs). These are the tolerability results even though the dose constraints adopted within the trial were more cautionary than the other prospective trials, with GTV Dmax hotspots < 130% vs. 150% in the Hilus trial, Dmax EQD2 to the PTB 81.9 Gy (the lowest within trials presented so far) and 5 mm PTV expansion. Another concern highlighted from the study is the risk of complications in patients undergoing thoracic invasive procedures after having received SBRT for UC lesions, mainly with regard to the PBT [30]. Such a trial highlights the importance of considering patients’ comorbidities at baseline and of following up with patients carefully even at a distance from treatment for the emergence of late severe adverse events.

On the other hand, completely positive data in treating UC lesions with SBRT come from the more recent SUNSET trial. It is a phase I study where the treatment dose for the first patients was established at 60 Gy/8 fx. Subsequent dosing might be, per protocol, escalated or de-escalated (60 Gy/5–6 fx or in 10–15 fx) according to toxicity outcomes (i.e., time-to-event continual reassessment method). After amendment only, the de-escalation option was allowed and, eventually, all 30 patients with primary NSCLC enrolled in the trial received the 60 Gy/8 fx schedule. Outcome data were comparable to the other studies reported: 3-year OS was 72.5%, PFS 66.1%, and LC 89.6%. Crude regional control (RC) and distant control (DC) rates were 96.4% and 85.9%, respectively. Toxicity data reported only two patients (6.7%) experiencing G3–5 adverse events related to treatment: one G3 dyspnea and one G5 pneumonia. Such important results were possible probably thanks to strict patient selection and dosimetry constraints: the PTV Dmax hotspot was limited to 120% and tumors with endobronchial invasion were excluded [29].

The most recent prospective data comes from the LUSTRE trial. This is the only phase III randomized trial including UC lesions reported so far, even though not specifically designed for UC tumors. The trial aimed to assess the superiority of SBRT (48 Gy/4 fx or 60 Gy/8 fx for central/UC lesions) vs. conventional radiotherapy (60 Gy/15 fx) in treating primary early-stage NSCLC. Even though not stratified for UC lesions specifically, but generically for peripheral vs. central/UC lesions, it included 23 (15%) UC lesions in the SBRT arm. So far, outcome data have been reported only for the whole SBRT arm population, with three-year LC at 87.6%, event-free survival (EFS) at 49.1%, and OS at 63.5%, but toxicity data were reported separately, with only one (4.3%) late (occurring at 12 months follow-up) G5 hemoptysis in a tumor abutting proximal bronchus. Overall, four (17.4%) G ≥ 3 treatment-related events in the UC SBRT sub-population were reported [28].

### 3.2. Survival Outcomes and Toxicity with SBRT for Ultra-Central Tumors: Retrospective Data

While prospective data mainly point to assessing the feasibility and toxicity of a 60 Gy/8 fx dose regimen, retrospective data have a higher variability with different impacts on survival and toxicity outcomes (Table 1).

Treatment regimens reported vary from 4 to 12 fractions and from 4.5 Gy to 13.75 Gy per fraction with survival outcomes roughly superposable with those of prospective data when accounted for common biases related to retrospective studies, some of them also including very few patients.

Some studies found worse local control for UC lesions when compared to analogously treated central lesions [39,54,61], while others did not [42,63]. A possible explanation for that could be the mean higher tumor volume for UC lesions and closer proximity to critical structures such as PBT or esophagus among others which could ultimately lead to a worse PTV dose coverage or lower total dose prescription as reported from the statistical analysis in some studies, where such parameters correlated with LC [33,39,56,58], even though in another study of 88 patients treated with IMRT or VMAT between 2008 and 2017, a BED10 ≥ 100 did not correlate with LC at univariate analysis [23].

With respect to toxicity, the rates of severe adverse events are variable and this may be due to different dose regimens used, reporting biases and small population numbers in some studies [31,32,44,45,59], but even within small studies important toxicity warnings are sometimes shown, with G ≥ parameters. Specifically, higher AE rates and three AE rates of 18% [38,41] and G5 events were confirmed within many studies even though sporadically.

The most common severe up to G5 AEs reported are respiratory (pneumonitis, hemoptysis, fistulae) and esophageal (fistulae), with higher rates reported according to lesion location, volume or specific dosimetry parameters. Specifically, higher AE rates and grades were found across many studies to be correlated with endobronchial invasion or close proximity to PTB rather than trachea [31,40,47,50], which is in accordance with prospective data reported. Other parameters linked to this concept reported to be correlated with worse toxicity outcomes are the GTV/ITV rather than the PTV [33,42,47] or Dmax to bronchi, lungs or esophagus rather than lungs V5 or V20 [33,38]. Notably, in the studies from Wang et al., including 88 patients, esophagus D2.5cc and D5cc were significantly correlated with the risk to develop G ≥ 3 esophageal events (*p* = 0.001 and 0.008, respectively) [23], whereas the main bronchus Dmean BED3 > 91 Gy increased significantly (*p* = 0.003) the risk of G ≥ 3 respiratory AEs in another study of 72 patients from Lodeweges et al. [52].

According to some studies, there seems to be a possible reporting bias risk in studies for SBRT in UC lung lesions with regards especially to cardiac toxicity, where some studies do not report severe cardiac SBRT-related toxicity, while non-cancer-related mortality is sensibly increased even several years after SBRT. A study from Ahmadsei et al. evaluated dosimetry to cardiac substructures in 60 patients who underwent SBRT for UC lesions. While only 3% G ≥ 3 AEs were reported, at a follow-up of 2 years, a 20% incidence of cardiovascular events (mainly valvulopathy and atrial fibrillation) was detected, with a positive correlation between dose to the pulmonary artery and superior cava vein and non-cancer-related deaths in this population [35]. Similarly, in a study of 49 patients treated with 50–60 Gy/5 fx from Iovoli et al., Dmax and Dmean to the sinoatrial node were found to be correlated with worse OS at cut-off values of 1309 and 836cGy, respectively. Moreover, in a multivariate analysis of another study of 83 patients treated with 50–55 Gy/5 fx, it was reported that D45% right atrium constraint (with candidate cutoff values of 890cGy) was significantly associated with non-cancer-related survival and overall survival (*p* = 0.026 and *p* = 0.011, respectively) [46].

Of note, there is a growing interest with regard to adaptive radiotherapy with MRI-based linacs, which enables optimal tumor tracking, permitting ablative doses with better-tailored dose distribution and less target uncertainty.

In 2023, Sandoval et al. reported a retrospective study of 38 patients treated with MRI-linac. In roughly half of cases, the prescribed dose was 60 Gy/8 fx, and efficacy outcomes were comparable with CT-based linacs, but with no acute toxicities and only 5% late G3 toxicities, with no differences between central et UC lesion treated from the same study [42].

Two more recent studies on MRI-linacs treatment were published this year with a total of 36 patients that were treated with 50 Gy/5 fx (as a median) or 60 Gy/8 fx, respectively. Both studies, even though numerically small, report no adverse acute or late G ≥ 3 events at a median follow-up of about 1.5 years. One study reported a PTB Dmax improvement of 5.7 Gy (59.4 Gy vs. 65.1 Gy) [32], and the other showed that the adaptive RT approach permitted a more adequate PTV coverage compared to the original plan [34].

A study of patients from prospectively maintained databases with MRI-linac was published in 2023 and included 16 UC patients who were treated at different dose levels ranging from 30–60 Gy in 5 fx, with the lowest dose levels prescribed to lesions abutting to esophagus. At a median follow-up of 24 months, LC was 93%, and one G3 esophagitis, one G3 bronchial bleeding and one G4 bronchial bleeding (in a patient receiving also VEGFR inhibitors) were reported [41].

## 4. A Proposal for Practical Workflow for Treatment of Ultra-Central Tumors

**Figure 1:**
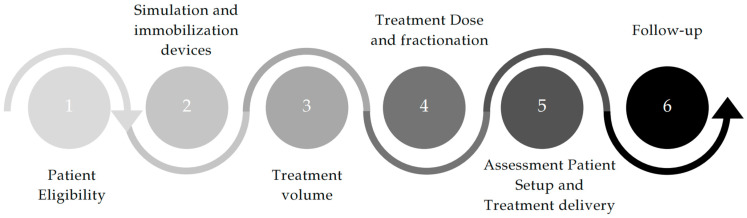
Treatment workflow of patients with ultra-central lung tumor.

The following sections will overview the general and technical requirements for the treatment of UC tumors. Each phase of the patient workflow in the radiotherapy department will be described and reviewed according to the available literature. This includes the patient’s eligibility for SBRT, the completion of treatment, and the subsequent follow-up. Figure 1 outlines the workflow of the topics covered in the next sections.

### 4.1. Patient Eligibility

Patient eligibility for lung SBRT follows specific indications, with no age-absolute contraindications [65]. Many studies have included and analyzed patients of a wide age range with no reported special concerns [18,27,51,52,53,56,57,58,59,60,63]. Historically, SBRT for lung lesions was recommended as a therapeutic option for a subset of patients who were not suitable for surgery due to medical comorbidities, anatomic limitations, or even patient refusal [66]. As per ESMO guidelines, postoperative morbidity and mortality can be assessed by defined models which have not been validated specifically for cancer patients. Indeed, it is mandatory to test cardiac and pulmonary function before planning surgical resection in order to estimate the risk of surgical morbidity [67]. The inoperability condition is also determined by poor lung function, which is evaluated using the following parameters: predicted forced expiratory volume in 1 s (FEV1) < 40%, predicted postoperative FEV1 < 30%, baseline hypoxemia (≤70 mmHg), hypercapnia (>50 mmHg), predicted reduced diffusing capacity < 40%, and predicted exercise capacity < 50%. Other severe comorbidities, such as severe pulmonary hypertension, diabetes mellitus with end-organ damage, severe cerebral, cardiovascular, peripheral vascular disease, or severe chronic heart disease, contribute to a condition of inoperability [2,68]. Unlike surgery, SBRT does not have a cut-off for lung function or other conditions, making it a viable therapeutic alternative even for patients with poor pulmonary function [68,69]. On the other hand, patients with an estimated life expectancy of <1 year, active systemic, pulmonary, or pericardial infection, and pregnant or lactating women were identified as contraindications for thoracic SBRT [2,6]. Conversely, eligible criteria for lung SBRT consisted of a centrally located inoperable primary lung cancer or metastasis from any other solid tumor [27]. Eligible patients should have a good performance status, defined as an Eastern Cooperative Oncology Group score of 0 to 2, and an early-stage tumor (T1-T3, N0, M0 as per the American Joint Committee on Cancer 8th edition staging) [29]. Regarding tumor size for defining the eligibility of patients for SBRT, a cut-off of 5 cm has been established by some authors [2,50,68]. However, more recently the recommended tumor size was extended to 6 cm [29]. 

### 4.2. Simulation Phase and Immobilization Devices

The simulation phase for SBRT of UC lung tumors must consider two critical issues: the tumor motion assessment and the patient immobilization strategy [70]. 

Regarding the need to evaluate tumor movement, the images required for simulation and planning often include detailed 4D CT scans. The 4D CT is adopted for analyzing the respiratory motion of the target with or without contrast enhancement. Its application in clinical practice for simulation scans of patients with UC cancer is often reported in the literature [27,29,49,51,52,53,54,55,57,58,59,60,63]. When this technology is not available, the tumor movement can be estimated by performing two CT scans, one on the inspiration and one on the expiration phase [71]. Patients typically also undergo three-dimensional (3D) CT acquisition, which may involve the use of intravenous contrast injection [49,55].

The choice of slice thickness depends on the protocol used and the type of CT scanner available. Normally, the slice thickness ranges between 2 and 3 mm [27,51,55,63]. The acquisition protocol generally extends from the lower mandibular margin to the lower hepatic margin and includes both lungs [59,71].

Regarding the immobilization device, SBRT already deviates from conventional treatments in the simulation phase and requires specific immobilization devices that cover a large part of the patient’s body both above and below the tumor [72]. Patients commonly undergo CT simulation in the supine position with arms above the head using a wing board, a specific device for thoracic treatment, a vac-lok bag or, in some centers, a thermoplastic body mask, and a knee and foot lock [73]. This setup optimizes reproducibility and minimizes motion or wobbling during radiotherapy. Despite the heterogeneity of different immobilization devices among centers, there is agreement in many studies regarding the use of vacuum cushions with other complementary devices in clinical practice for the treatment of UC pulmonary lesions [27,50,51,53,54,55,57,63]. Patients with upper mediastinal lymph nodes targeted were immobilized in an extended thermoplastic head and shoulder mask with their arms in the down position along their body [53,57]. Active motion management techniques, such as breath-holding techniques and abdominal compression, are often not used or described in most studies due to reduced motion of UC lesions and therefore are not broadly recommended. Regarding abdominal compression, this device is not used as an immobilization device in all studies and also not in all RT centers. For example, in the study by Lindberg et al., it is reported that only four out of nine participating centers used this device [27]. Notably, abdominal compression is employed under specific conditions; for instance, Nguyen et al. used it to limit diaphragmatic excursion to ≤1 cm [63]. In addition, Regnery et al. used the device if the tumor was located in a lower lung lobe [54]. The study by Giuliani et al. highlighted the need for abdominal compression in tumors with more than 1 cm of motion [29]. It has also been used in other studies [50,57]. It is important to emphasize that the choice of immobilization device depends on the protocol, availability, and internal experience of each institution. 

The use of deep-inspiration breath-holding is rare for UC lung lesions. In fact, Zhao et al. reported that this technique was systematically not used [58], and Breen et al. noted that it was employed in only 10% of cases [51]. Mihai et al.’s study highlighted the use of the breath-holding technique only when tumor motion exceeded 5 mm in any direction [53]. 

**Figure 2:**
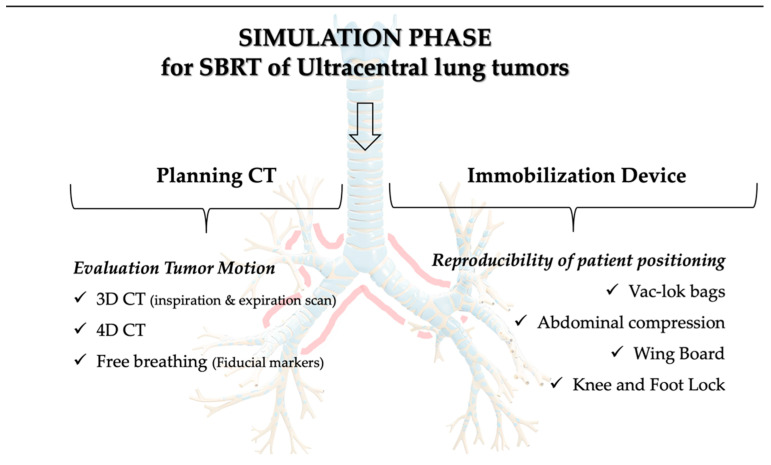
The simulation phase for SBRT planning of Ultra-central Lung tumors.

Based on the included studies, Figure 2 summarizes the modalities of CT planning acquisition and the immobilization devices used in patients with UC lung tumors.

### 4.3. Treatment Volume

From the analysis of the included articles, it emerges that the methodology used for the delineation of treatment volumes is heterogeneous. Starting with the gross tumor volume (GTV), it is evident that this can be delineated on the 3DCT acquired using a lung window [55,56] and with the aid of co-registered images obtained from contrast-enhanced CT and PET-CT scans [60]. Alternatively, GTV can be defined using 4D CT images by outlining it on both inspiratory and expiratory scans [29,57] in the end-expiration phase [53] or by delineating it using the average phase of the 4D CT [58]. In patients treated with breath-holding techniques, GTV is contoured on CT scans acquired during the breath-holding phase [53].

Most of the included studies agree on the definition of an interal target volume (ITV) and describe its creation by expanding the GTV contour to encompass tumor shifts across all respiratory phases of the 4D CT linked with the breathing cycle (35, 39, 40, 45, 57, 58, 60, 66]; therefore, the 4D CT scan plays a crucial role in defining tumor motion and allows delineation of the ITV contour during all phases of respiration [74]. In lung SBRT practice, it is not common to add a margin from the GTV to the clinical target volume (CTV) [51,53,68,70]. However, in some studies, a CTV is defined by adding a 2–5 mm margin to the GTV-ITV [54,75]. 

Finally, to create the planning target volume (PTV), a margin was added to the ITV to account for set-up uncertainties. The isotropic growth to create the PTV was generally around 5 mm [18,29,49,51,55,56,57,58,59,60]. Sometimes the isotropic margin added from the ITV to the PTV was reduced up to 3 mm [52,53,55] or the longitudinal (cranio-caudal) margin was increased [60]. In many centers that participated in the Lindberg et al. study, the margin was widened up to 10 mm [27]. In the breath-holding technique, the GTV was defined on the CT images acquired while holding breath, the creation of the ITV was not mandatory and the PTV was created by adding an isotropic margin of 5 mm to the GTV [53,68].

The recommended OARs to be contoured in the UC lung tumor are the following: spinal cord, trachea, lungs, heart, esophagus, proximal bronchial tree, brachial plexus and great vessels (ascending aorta, vena cava superior, pulmonary artery and pulmonary vein) [27,29].

### 4.4. Treatment Dose and Fractionation

An overview of the treatment dose and fractionation used in each study included is reported in Table 1. While older retrospective data may vary in terms of fractionation and doses, the most recent retrospective studies and prospective data aimed to assess the feasibility of 60 Gy in eight fractions, with alternate results, but most recently reassuring data. Overall data show that when adequately selected and planned, this fractionation regimen seems to provide high local control rates with acceptable toxicity profiles [29,58,59,61]. High caution and probably a dose de-escalation should be used when treating tumors with endobronchial invasion or abutting PTB or esophagus, combined with a careful long-term follow-up and benefit/risk evaluation for later thoracic invasive procedures (i.e., bronchoscopy) [30,41,42]. Moreover, it seems that limiting PTV Dmax ≤ 110–120% helps in lowering the toxicity rates [29,31,32].

### 4.5. Setup and Motion Management Systems

A comprehensive image guidance and motion management strategy needs to be applied and maintained with sufficient technology and procedures to ensure safe and effective positioning and mitigate motion-related errors [76]. Depending on the equipment used in clinical practice, various technologies can be employed to assess patient positioning and verify tumor motion during treatment. Based on the study by Caillet et al. [73], it is possible to recognize the image-guided RT (IGRT) strategies and motion management systems employed in the room prior to the treatment delivery and those that are used during the treatment. 

According to most studies, prior to treatment, daily imaging verification is performed to assess patient positioning and correct any potential setup errors [29,51,52,53,55,57,58,59,77]. In recent years, studies have highlighted the adoption of kV cone-beam-computed tomography (kV-CBCT) as a key modality for IGRT acquisition with online correction performed by multidisciplinary teams [29,51,52,53,55,57,58,59,77]. In particular, the use of 3D-IGRT modalities, such as kV-CBCT, is preferable to 2D techniques like electronic portal imaging devices. kV orthogonal imaging offers a more comprehensive evaluation of patient deformation and rotation, showing the internal anatomy of the patient and providing higher contrast visibility of soft tissues [73,78,79]. The main limitation of 3D kV-CBCT for lung imaging is that it averages projections from different respiratory phases to produce a single 3D scan, which can lead to blurred areas and provide incorrect information about the actual tumor amplitude and its relative position during the respiratory phases [73,80,81]. The 4D CBCT provides daily motion data resulting in precise information about the tumor’s trajectory on that day. This helps to maintain tighter margins around the target and reduces inter-observer variability in patient positioning [73,82]. 

In addition, for optimal alignment correction, it is important to use a six-degree-of-freedom robotic couch, which makes it possible to correct not only translation but also rotational displacements, which are assessed during IGRT to account for the patient’s internal movement and rotation, as in the study by Shahi et al. [57,73,83]. Additionally, patient positioning can be evaluated using an optical system that registers and correlates the patient’s surface. In recent years, multiple optical systems have become commercially available, and these devices are increasingly being used for interfraction setup in SBRT lung tumor treatments [73,84,85]. Moreover, the major advantage of the use of optical systems is the control and detection of the intrafraction patient motion during treatment delivery, without the use of ionizing radiation or invasive procedures [84,86]. In addition, the optical system can automatically interrupt the beam, interfacing with the linear accelerator, if the patient moves beyond a predefined tolerance threshold [87]. In SBRT performed with the DIBH technique, the optical system is used to guide patients in performing deep voluntary inspirations with reproducibility and to provide visual feedback to ensure accuracy [88]. Breathing control methods also include immobilization devices, as previously mentioned, as well as those that use abdominal compression. These devices mechanically limit abdominal motion during respiration and can be applied in various ways [73]. 

In the study by Guillaume et al., Cyberknife is used in high percentage for the treatment of UC lung tumors [49]. Real-time tumor tracking by the placement of markers in or near the tumor is often adopted to treat patients with the Cyberknife system [87]. The markers can be placed by different approaches depending on the risk of complication such as pneumothorax: via the percutaneous, intra- or extra-pulmonary, or via the vascular approach [89,90]. Percutaneous marker placement typically relied on fluoroscopy or CT guidance to position platinum markers either within or near the tumor (intrapulmonary technique) or on the thoracic wall adjacent to the ribs (extrapulmonary technique). The vascular method entailed deploying embolization coils into small subsegmental branches of the pulmonary artery near the tumor via a catheter in order to reduce the risk of complications related to the insertion procedure, particularly pneumothorax [90]. Commonly, at least three markers are inserted to correct for translational and rotational target motions. The motion of implanted markers is usually detected through a study of orthogonal X-ray images performed during breathing in order to build a respiratory model correlated with external positional information extrapolated from infrared detectors [91].

### 4.6. Follow-Up

The follow-up protocols of the various studies on UC lung SBRT show some similarities, particularly with regard to timing, imaging methods and toxicity assessment. In most studies, clinical follow-up and imaging examinations were performed every 3 months, especially in the first year after treatment [18,27,29,49,50,53,55,59,60,63,77]. For the second year, follow-up examinations were usually scheduled every 3 to 6 months, followed by semi-annual or annual check-ups in the subsequent period [18,27,29,49,53,54,55,57,58,59,60,63].

In terms of imaging modality, CT scans of the chest with or without contrast were found to be the main imaging modality used to monitor disease progression and response to treatment [27,29,49,51,52,54,58,59]. In addition to chest imaging, some studies included broader imaging protocols, such as CT scans of the abdomen and pelvis or brain imaging with CT or magnetic resonance imaging (MRI) to assess possible distant metastasis [49,53,59]. 

Almost all studies included additional imaging such as PET-CT in the follow-up protocol if CT findings were indeterminate or suggestive of progression [18,27,29,49,50,51,53,54,55,58,59,60,77]. The use of the Response Evaluation Criteria in Solid Tumors (RECIST) version 1.0/1.1 to evaluate tumor response was consistent across several studies [18,27,29,49,54,56,57,58,59,77].

Toxicity was graded according to the Common Terminology Criteria for Adverse Events (CTCAE) version 4.0/4.03 [18,27,29,55,56,59,60,63,77] or 5.0 grading system [51,52,54,57,58]. In addition to radiological examinations and physical examinations, electrocardiography and pulmonary function tests can also be carried out during follow-up care [18,27,29,54].

## 5. Conclusions

To the best of our knowledge, this is the first overview of the treatment workflow of patients treated with SBRT for UC lung tumors. By providing a critical review of dedicated literature and a “roadmap” on the general and technical requirements for prescription, motion management, patient selection and dosimetric constraints, it could contribute to SBRT being administered safely and effectively for UC lung tumors among centers with different expertise. Nonetheless, there are limitations to be considered when evaluating our results and suggestions. First, it should be noted that we did not conduct a systematic review or meta-analysis, which prevented us from performing any statistical analysis other than descriptive. Our results highlight how the role of SBRT in this setting is still an unanswered question. Indeed, the literature is still sparse, contradictory, and mostly based on retrospective data; therefore, any systematic analysis would not provide robust additional data. Second, we are aware that our workflow proposal may be modified according to the expertise, practice routine and resources of various centers; nonetheless, we still wanted to provide an evidence-based model that could be broadly shared to improve and counsel such challenging treatments. Further prospective and randomized studies are awaited in order to provide high-quality data and strong evidence on the optimal SBRT technique, schedule and dosimetry constraints.
